# Group II intron and repeat-rich red algal mitochondrial genomes demonstrate the dynamic recent history of autocatalytic RNAs

**DOI:** 10.1186/s12915-021-01200-3

**Published:** 2022-01-07

**Authors:** Dongseok Kim, JunMo Lee, Chung Hyun Cho, Eun Jeung Kim, Debashish Bhattacharya, Hwan Su Yoon

**Affiliations:** 1 Department of Biological Sciences, Sungkyunkwan University, Suwon, 16419 South Korea; 2 Department of Oceanography, Kyungpook National University, Daegu, 41566 South Korea; 3 Department of Biochemistry and Microbiology, Rutgers University, New Brunswick, NJ 08901 USA

**Keywords:** Genome expansion, Group II introns, Repeated sequences, Horizontal gene transfer, Red algae

## Abstract

**Background:**

Group II introns are mobile genetic elements that can insert at specific target sequences, however, their origins are often challenging to reconstruct because of rapid sequence decay following invasion and spread into different sites. To advance understanding of group II intron spread, we studied the intron-rich mitochondrial genome (mitogenome) in the unicellular red alga, *Porphyridium*.

**Results:**

Analysis of mitogenomes in three closely related species in this genus revealed they were 3–6-fold larger in size (56–132 kbp) than in other red algae, that have genomes of size 21–43 kbp. This discrepancy is explained by two factors, group II intron invasion and expansion of repeated sequences in large intergenic regions. Phylogenetic analysis demonstrates that many mitogenome group II intron families are specific to *Porphyridium*, whereas others are closely related to sequences in fungi and in the red alga-derived plastids of stramenopiles. Network analysis of intron-encoded proteins (IEPs) shows a clear link between plastid and mitochondrial IEPs in distantly related species, with both groups associated with prokaryotic sequences.

**Conclusion:**

Our analysis of group II introns in *Porphyridium* mitogenomes demonstrates the dynamic nature of group II intron evolution, strongly supports the lateral movement of group II introns among diverse eukaryotes, and reveals their ability to proliferate, once integrated in mitochondrial DNA.

**Supplementary Information:**

The online version contains supplementary material available at 10.1186/s12915-021-01200-3.

## Background

Group II introns are widely distributed in the organelle genomes of plants, fungi, algae, and protists, and also invade the genomes of bacteria and viruses [1, 2]. Because group II introns are mobile genetic elements that have the ability to insert at specific target sequences [3], homologous group II introns have spread into the same site in organelle genomes of distantly related species [1]. Despite knowledge of the mechanisms of spread, group II intron origins are often challenging to study because of rapid sequence decay following invasion and spread into different sites [4]. To this end, conserved group II intron-encoded proteins (IEPs) have been widely used to trace their origins [5, 6]. Generally, group II intron IEPs consist of four sequence domains encoding reverse transcriptase (RT), maturase (X), DNA binding (D), and DNA endonuclease (En) functions [7]. Domains such as RT and D are usually essential for retro-transposition, but they are not necessary for splicing. Therefore, mutations in RT and D post-invasion have little effect on the host, and some group II introns can survive for extended periods, in particular, if the splicing process is co-opted by the host to regulate gene expression or other functions [8].

The life-cycle of group II introns comprises three well-characterized phases [9]: invasion of the target site, sequence drift/divergence, and deletion. Post-deletion, empty target sites may be invaded by other cognate introns and this cycle can repeat itself. The phylogenetic distribution of mobile group II introns suggests that they have originated from bacteria and were transferred to eukaryotes *via* the two primary endosymbioses that led to the origins of mitochondria and plastids [6, 10]. This idea is supported by the presence of IEP-containing group II introns in most bacterial genomes that act as retroelements with functional ribozyme and RT components [11]. In contrast, organelle group II introns often lack open reading frames (ORFs) and/or contain degenerate IEPs or RNA structures [7, 12]. Although unproven, group II introns are considered the ancestors of spliceosomal introns found in many eukaryotes, including humans. This idea is supported by the common splicing mechanism and structural similarity between group II introns and small nuclear RNA (snRNA)/intron/exon pairing that comprises the spliceosome during the splicing reaction. Group II introns are also associated with nuclear non-long terminal repeat (LTR) retroelements. In fact, RTs of non-LTR elements are phylogenetically closely related to IEPs in group II introns [1, 6]. Group II introns are believed to have existed in the nuclear genome of early eukaryotes. Their disappearance may be explained by RNA instability and effects on translation, or the possibility that they were co-opted to give rise to canonical spliceosomal introns [13].

The Porphyridiophyceae is a mesophilic unicellular red algal class, which inhabits sites with varying salinity including freshwater, brackish water, and seawater. These red algal taxa contain many sulfated cell wall polysaccharides as antioxidants to provide protection against reactive oxygen species (ROS) [14]. The nuclear genome of *Porphyridium purpureum* CCMP1328 has been used as a model to study horizontal gene transfer (HGT) and phycobilisome evolution [15, 16]. The plastid genome of *P. purpureum* CCMP1328 contains an exceptionally high number of introns (43 introns – one group I and 42 group II introns) with unusual twintrons [17–19]. However, *Porphyridium* mitochondrial genomes (hereafter, mitogenomes) have not yet been studied. To advance understanding of autocatalytic RNA evolution, we generated mitogenomes from three *Porphyridium* isolates: *P. purpureum* CCMP1328, *P. purpureum* SAG1380-1a, and *P. aerugineum* CCMP1948. Because these mitogenomes are unusually large in size, we focused on the role of group II introns and repeats in mitogenome growth.

## Results and discussion

### General features of *Porphyridium* mitogenomes

**Fig. 1:**
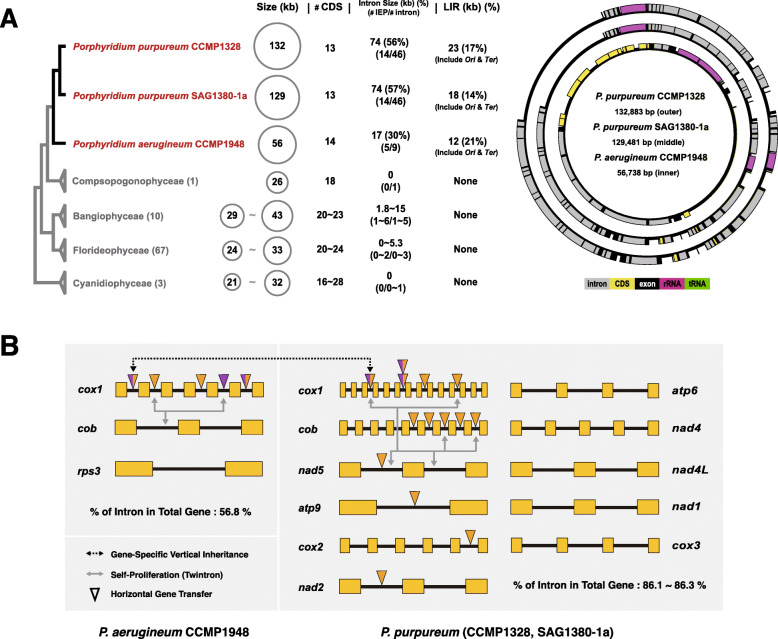
Schematic comparison of red algal mitogenomes. **a** The characteristics of three *Porphyridium* mitogenomes (mitogenome size, the number of intron (IEPs), the length of LIR) are shown, when compared to other red algae. The mitochondrion genome maps in the right panel show *P. purpureum* CCMP1328, *P. purpureum* SAG1380-1a, and *P. aerugineum* CCMP1948 from the outside-in. Intron, CDS, exon, rRNA, and tRNA are marked in different colors in these maps (gray, yellow, black, red, and green, respectively). The phylogenetic tree in the left panel is based on a previous study [20]. **b** All genes with introns present in *Porphyridium* mitogenomes are shown. The origins of the IEPs present in each intron are represented using triangles of different colors (fungi-related: purple-colored; stramenopiles-related: orange-colored; both-related: mixed with purple- and orange-colored). The movement of the IEPs and twintrons are shown using arrows (gene-specific vertical inheritance: dotted, black-colored arrow; self-proliferation: gray-colored arrow)

The complete circular mitogenome sequences of *P. purpureum* CCMP1328 (MT483996), *P. purpureum* SAG1380-1a (MT483997), and *P. aerugineum* CCMP1948 (MT483995) show significant size variation (132 kbp, 129 kbp, and 56 kbp, respectively). These mitogenomes are 3-fold larger in size than any of the 81 currently available red algal mitogenomes that are 21~43 kb in size (Fig. 1a and Additional file 2: Table S1). *Bangia fuscopurpurea* (NC_026905, Bangiophyceae), the previously largest reported mitogenome (43,517 bp), includes two tandem repeats and five introns (two in the large subunit rRNA gene and three in the *cox1* gene). The *Porphyridium* mitogenomes, however, encode fewer protein coding genes (i.e., 13–14 CDS) than other red algae (i.e., 16–28 CDS), containing two subunits of the ATP synthase complex (*atp*6 and *atp*9), one subunit of cytochrome-b (*cob*), three subunits of cytochrome-c oxidase (*cox1*, *cox2*, and *cox3*), and seven subunits of NADH dehydrogenase (*nad1*, *nad2*, *nad3*, *nad4*, *nad4L*, *nad5*, and *nad6*). There is one partial small ribosomal subunit (*rps3*) in *P. aerugineum* CCMP1948 (Additional file 2: Table S2). With two ribosomal RNAs (*rnl* and *rns*), *P. purpureum* CCMP1328, *P. purpureum* SAG1380-1a, and *P. aerugineum* CCMP1948 contain 13, 13, and 9 tRNA genes, respectively. In contrast, these genomes have a high intron content (i.e., 46, 46, and 9 introns) with 14, 14, and 5 IEPs in *P. purpureum* CCMP1328, *P. purpureum* SAG1380-1a, and *P. aerugineum* CCMP1948, respectively, that explain the increase in genome size (Fig. 1).

Correlation analysis shows that introns (*r*^2^ = 0.992) and intergenic regions (*r*^2^ = 0.967) are major factors in genome size expansion (Additional file 1: Fig. S1a). In fact, introns in these three taxa account for 86.3%, 86.1%, and 56.8% of the total genic region, respectively. As an example, the *cob* gene of *P. purpureum* CCMP1328 is 20,224 bp in size including 1150 bp of exon and 19,074 bp (94.3%) of intron sequences (Additional file 2: Table S3). The intergenic regions between CDSs (including ORFs) also comprise a large proportion (27~36%) of the total length of mitogenomes. Based on correlation analysis, two major contributors to mitogenome expansion are a total of 74 kbp (56% of mitogenome size) of intron sequences including 14 IEPs and 66 tandem repeats concentrated in the large intergenic region (LIR: 23 kbp, 17%) of *P. purpureum* CCMP1328. Likewise, the mitogenome of *P. aerugineum* CCMP1948 has 17 kbp (30% of mitogenome size) of introns including five group II IEPs and 47 tandem repeats in the LIR (LIR: 12 kbp, 21%). This massive expansion in mitogenome size has also been reported in the alpine soil green alga *Chlorokybus atmophyticus* (201,763 bp). It is interesting to note that the *C. atmophyticus* mitogenome also includes a large number of introns (i.e., six group I introns and 14 group II introns), 249 tandem repeats, and extended intergenic regions rich in repeated elements. Interestingly, 10 group II introns in the *C. atmophyticus* mitogenome are located in tRNA genes [21]. Therefore, introns (30–56%) and LIRs (17–21%) including many repeats, are the major factors underlying organelle genome size expansion.

We compared tandem repeat distribution among red algae (Additional file 1: Fig. S2b) and found that the Porphyridiophyceae has the largest number of mitogenome-encoded tandem repeats. Interestingly, *P. aerugineum* CCMP1948 has the greatest tandem repeat content but the smallest mitogenome among the Porphyridiophyceae. In contrast, *P. purpureum* CCMP1328 has more tandem repeats and larger mitogenomes than *P. purpureum* SAG1380-1a. To compare the repeat density in each species, we counted the number of repeats per 1,000 bp (Repeat density; Additional file 1: Fig. S2c). The repeat density in *Galdieria sulphuraria* was 2.05 per 1 kbp, which is the highest among all mitogenomes of red algae. The average repeat density of *Porphyridium* (0.95 repeat/kbp) was smaller than in Cyanidiophyceae (1.23 repeat/kbp), suggesting that the repeats in *Porphyridium* are concentrated in the large intergenic regions (i.e., *P. purpureum* CCMP1328: 2.85 repeat/ kbp; *P. purpureum* SAG1380-1a: 3.24 repeat/kbp; *P. aerugineum* CCMP1948: 3.70 repeat/kbp). Additionally, the three *Porphyridium* taxa contain larger tandem repeats (2–500 bp) than those of 10 bangiophycean species (2–100 bp) and 15 florideophycean taxa (2–300 bp) (Additional file 1: Fig. S2d).

### Accumulation of repeats in the large intergenic region

**Fig. 2:**
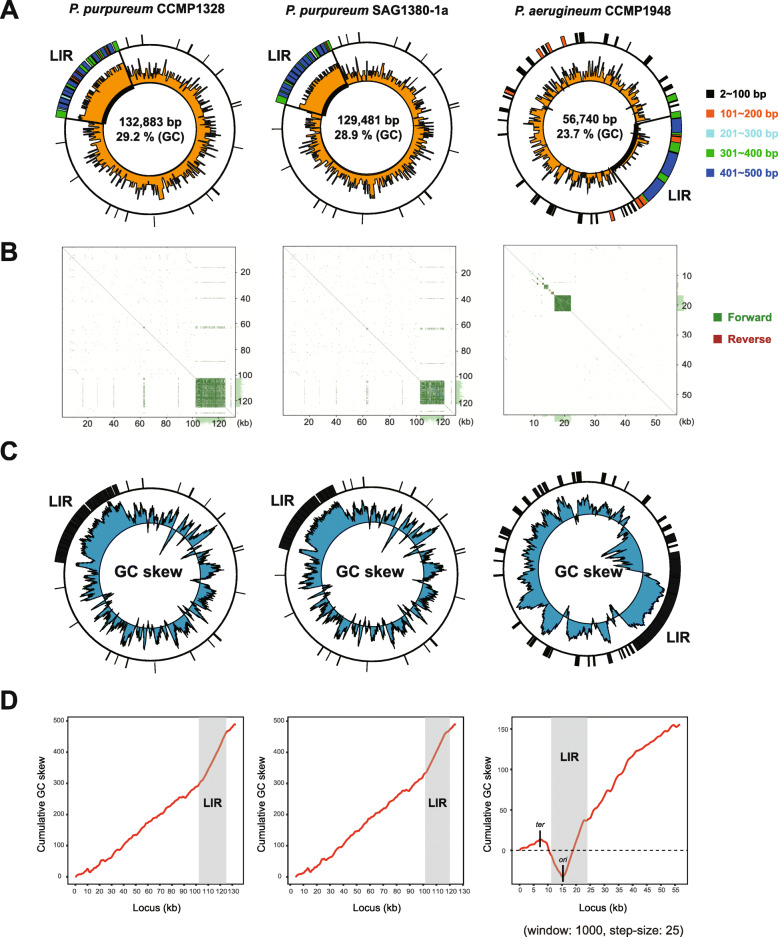
Repeat and GC content analysis of *Porphyridium* mitogenomes. **a** The distribution of repeats by length is shown in the outer circle, whereas GC content is shown in the inner circle. LIR is the large intergenic region. **b** Three YASS dot-plots generated using self-comparisons. The green segments show alignments of forward reads and red segments show alignments between the reverse complement of one sequence and the forward read of the other. **c** Three GC skew plots are shown, each obtained from the mitogenome of *Porphyridium* species with the distribution of tandem repeats (window size = 1000, step size = 25). **d** Cumulative GC skew plots of the three studied *Porphyridium* species. Sequence corresponding to the large intergenic region (LIR) is indicated in gray

Analysis of genome similarity using the YASS program [22] shows that the most repeats are located in large intergenic regions (LIRs) (i.e., 23 kbp, 18 kbp, and 12 kbp, in CCMP1328, SAG1380-1a, and CCMP1948, respectively, see Fig. 2a). Mitogenome alignments highlight the repeat-rich LIRs in all three *Porphyridium* species (Fig. 2b). These tandem repeats range in size from 2 to 500 bp. Repeats corresponding to 2–100 bp are the most abundant in rRNA, intergenic, intron, and exon regions in all three species (Additional file 1: Fig. S2a). Tandem repeats > 100 bp in size are distributed primarily in intergenic regions.

Repeats are present in all genomes and are particularly abundant in eukaryotes. They exist in both coding and noncoding regions and show length variation through the addition or deletion of sequences. The replication slippage model, known as copy-choice recombination, accounts for repeat length variation [23]. Replication slippage is generally caused by partial denaturation and displacement processes in a DNA strand, causing mispairing at complementary bases at sites where short tandem repeats exist. In the process of replication or repair of mispairing, insertion or deletion of short repeat units occurs, and as a result, tandem repeats of various lengths are generated [24]. Mutations and rearrangements caused by misalignment during DNA replication are sources of genetic diversity that frequently occur in prokaryotes and eukaryotes. In repetitive DNA sequences, dislocation between the replication strand and its template can occur. Repeat misalignment occurs over a range of lengths, from a few to hundreds of base pairs and generally occurs at sites close to the origin of replication [23, 25–27]; these replication slippages are primarily found near the site of replication origin [25, 28, 29]. Unusually large LIRs have been found in several plant mitogenomes [30–32], but their origin(s) remain unclear [32].

The cumulative GC skew diagram, calculated as (G−C)/(G+C), can be used to predict the origin of replication and terminus [33]. GC contents of the three *Porphyridium* mitogenomes (CCMP1328, SAG1380-1a, CCMP1948) were 29.2%, 28.9%, and 23.7%, respectively (Fig. 2a). However, GC contents in the LIR were significantly higher in *P. purpureum* CCMP1328 (39.6%) and *P. purpureum* SAG1380-1a (39.8%). In contrast, GC content of the LIR (20.6%) in *P. aerugineum* was lower than the average (23.7%). All three species have high GC skew in the LIR (Fig. 2c), and cumulative GC skew analysis shows that the slope of these values within the LIR region was the highest in two *P. purpureum* strains (Fig. 2d). In contrast, *P. aerugineum* CCMP1948 showed one maximum peak and one minimum peak within the LIR. Interestingly, among the three *Porphyridium* species, two peaks were identified only in *P. aerugineum* CCMP1948, suggesting the replication origin and terminus, and the minimum peak corresponding to the replication origin was found in the LIR of this species. From these results, we infer that a large number of repeats in the LIR of *P. aerugineum* CCMP1948 were caused by DNA slippage at the site of origin of DNA replication. In contrast, there were no distinct peaks in the two strains of *P. purpureum*, and the slope of the cumulative GC skew increased markedly in the LIR (GC content is 39.3% and 39.7% in CCMP1328 and SAG1380-1a, respectively). The origin of replication in the yeast nuclear genomes is generally a short, intergenic region that contains A/T-rich DNA. In contrast, replication origins in metazoan and plant nuclear genomes are long and G/C-rich [34]. In the case of the *Arabidopsis thaliana* nuclear genome, the region ±0.1 kb from the replication origin midpoints has a much higher GC content (44.5%) than other regions (35%) [35]. Because of a close phylogenetic relationship between *P. sordidum* and *P. purpureum*, and an earlier-divergence of *P. aerugineum* within the genus *Porphyridium* [36], we suggest that the replication origin of *P. purpureum* is located within the LIR. We could not however detect two peaks in the LIRs using the cumulative GC skew method. Added evidence for our hypothesis is the extended G/C-rich region in the LIR of many plant species [35]. Therefore, we postulate that the two *P. purpureum* strains are likely to have an analogous repeat expansion caused by DNA slippage at the replication origin, as in *P. aerugineum.*

### The origin and expansion of group II intronic ORFs

The mitogenomes determined in our study contain multiple intronic ORFs: i.e., 14 in CCMP1328, 14 in SAG1380-1a, and five in *P. aerugineum*. The intronic ORFs in CCMP1328 and SAG1380-1a are located in the *cob* (5), *cox1* (5), *atp9* (1), *cox2* (1), *nad2* (1), and *nad5* (1) genes, whereas the five in *P. aerugineum* are all encoded in *cox1* (Fig. 1b and Additional file 2: Table S3). Based on a BLASTx search (*e*-value cutoff = 1.0*e*^−5^) against the NCBI non-redundant database, all *Porphyridium* intronic ORFs are intron-encoded proteins (IEPs) in group II introns. Group II introns can be classified into several types based on RNA sequence and secondary structure. Alternatively, group II introns can be classified according to phylogenetic analysis of their IEP amino acid sequences. These two classification methods can be used for different purposes. The classification method based on RNA sequence and secondary structure can be applied to all introns regardless of whether the group II intron encodes an IEP. In contrast, IEP-based classification is more specific and suitable to describe the origin of group II introns corresponding to particular phylogenetic clades [6]. For this reason, we used IEP-based phylogenetics to determine the origin of group II introns [5]. Particular attention was paid to the relationship between nucleus and organelle-encoded IEPs due to endosymbiotic gene transfer (EGT) between these compartments, or alternatively, acquisition from bacteria or other eukaryotes *via* horizontal gene transfer (HGT). Amino acid sequences of the 33 IEPs were used as queries to search for homologs (*e*-value cutoff = 1.0*e* ^-5^) in the nuclear and plastid genomes of *Porphyridium* species (PRJNA560054, MF401423.1, unpublished genome data of *P. purpureum* SAG1380-1a and *P. aerugineum* CCMP1948). This analysis identified 26 plastid IEPs, whereas no hits were found in the nuclear genome. Protein similarity network analysis suggests a possible evolutionary link between mitochondrial and plastid IEPs (Additional file 1: Fig. S3a).

**Fig. 3:**
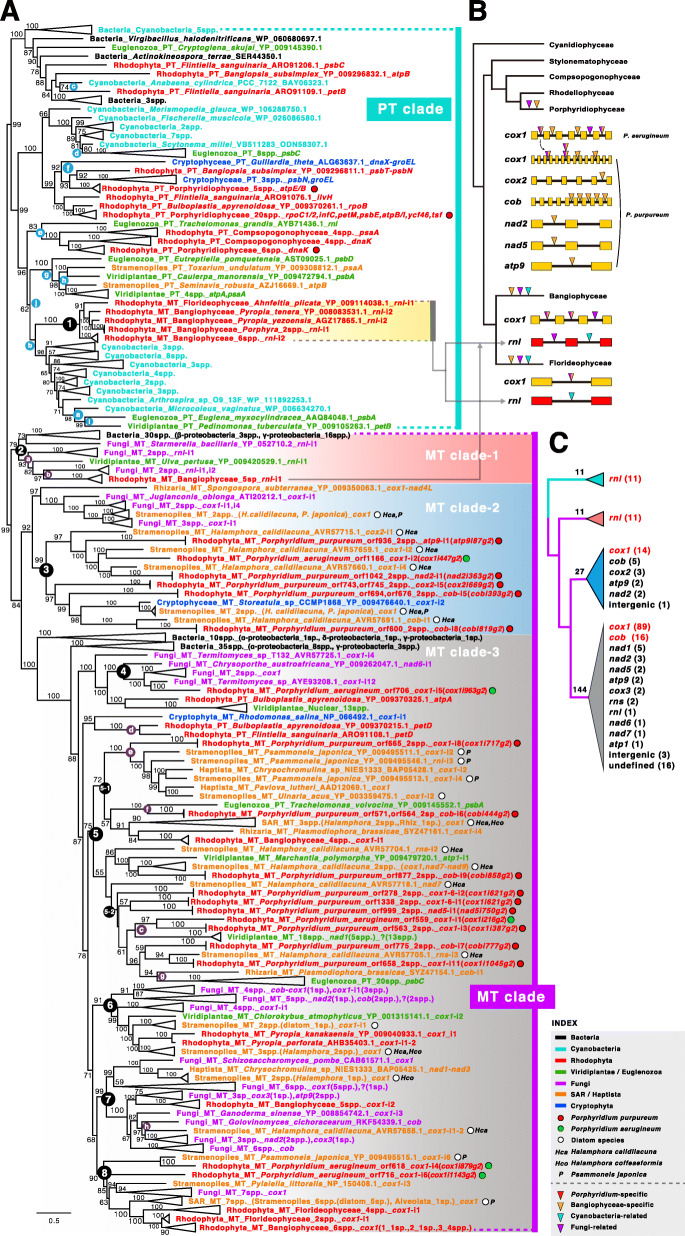
Maximum likelihood phylogeny of group II intron ORFs. **a** Maximum likelihood tree of group II intronic ORFs was identified using BLASTp (*e*-value cutoff = 1.0*e*^−5^). The taxa corresponding to red algal mitochondria are marked in red. The taxa corresponding to red algal plastids are marked in blue. Among the red algal taxa, *Porphyridium* species are marked with a colored circle (*P. purpureum* MT; red circle, *P. aerugineum* MT; green circle, *Porphyridium* PT; red circle). The taxa corresponding to fungi are in purple, cyanobacteria are in cyan, non-cyanobacterial bacteria are in black, non-red algal mitochondria are in orange, and non-red algal plastids are in green text. The clades described in the main text are indicated by black-filled and white-numbered circles. The clades corresponding to the contents in Fig. 5a are presented in colored circles in alphabetical order. The location of the intron (i.e., *rnl*-i1 = intron 1 in *rnl*) is shown after the gene name. We used the previously proposed nomenclature [37] to identify the *Porphyridium* mitochondrial introns. Bootstrap values below 50% were removed. **b** The distribution of red algal mitochondrial group II introns based on the phylogenetic tree. The origins of IEPs present in each intron are shown using triangles of different colors (fungus-related: purple; stramenopiles-related: orange; both-related: mixed with purple and orange). **c** A simplified tree indicating the number of mitochondrial genes with group II introns based on clades (PT clade and MT clades 1, 2, and 3)

To identify putative origins *via* HGT, we used blast with the 33 mitochondrial and 26 plastid IEPs encoded in *Porphyridium* group II introns to query the NCBI database (search cutoff, *e*-value ≤ 1.0*e*^−5^). This search returned sequences from 424 taxa including diverse bacteria and eukaryotes (rhodophytes, viridiplants, rhizarians, stramenopiles, cryptophytes, haptophytes, euglenozoids, and fungi). The maximum likelihood tree constructed from these data (Fig. 3a) identified two major clades (PT [plastid including cyanobacteria] and MT [mitochondria including bacteria]) that were likely derived from cyanobacteria and alpha-proteobacteria, respectively. Interestingly, mitochondrion encoded *rnl* group II IEPs of Bangiophyceae (8/10 reported MTs; *Pyropia tenera*, *P. yezoensis* (2), *P. nitida*, *P. perforate*, *P. haitanensis*, *Porphyra umbilicalis*, *Por. purpurea* (2), and *Bangia fuscopurpurea*) and Florideophyceae (i.e., *Ahnfeltia plicata*) were positioned within the PT clade with cyanobacteria (clade-1 in PT clade). Therefore, group II IEPs may have been transferred from unspecified cyanobacterial donors into the *rnl* gene of the ancestor of Bangiophyceae, followed by an independent transfer to *Ahnfeltia*, because only *A. plicata* contains *rnl* group II IEPs among 67 reported florideophycean mitogenomes. Because there are no group II IEPs in the plastid genomes of Bangiophyceae and Florideophyceae, it is unlikely to have been transferred between organelles within a cell (i.e., from plastid to mitochondria or *vice versa*). Because these cyanobacterium-derived mitochondrial group II IEPs in the *rnl* do not cluster with other red algal plastid group II IEPs, it is unlikely that they were vertically inherited from the early-diverged plastid genomes (i.e., Cyanidiophyceae, Porphyridiophyceae, Compsopogonophyceae, Rhodellophyceae). Evidence for the introduction of group II intronic ORFs from cyanobacteria to mitochondria exists for a red (*rnl* in *Porphyra purpurea*) [38] and a brown alga (*rnl* in *Pylaiella littoralis*) [39].

Within MT, three distinct clades were found (MT clade-1, -2, and -3). MT clade-1, comprises 30 bacteria (including beta and gamma-proteobacteria), five fungi, one green alga, and five bangiophycean red algae, and is positioned in *rnl* genes that differ from cyanobacterium-derived *rnl* group II IEPs (clade-1 in the PT clade, see above). Although both are located in the same mitochondrial *rnl*, this result suggests that these two types of *rnl* group II IEPs of the Bangiophyceae have different origins (i.e., independent HGTs from cyanobacteria and proteobacteria, respectively, see Fig. 3b). The MT clade-3 consists of two early diverged proteobacterial clades and diverse eukaryotes (i.e., red algae, green algae,stramenopiles, haptophytes, cryptophytes, rhizarians, and fungi) and various mitochondrial genes (i.e., *cox*, *cob*, *nad*, *atp*, *rnl*, and *rns*) (Fig. 3c and Additional file 2: Table S4). Most of the group II IEPs are located in mitochondrial genes in MT clade-3; however, this clade also includes several plastid-encoded group II IEPs (i.e., *Bulboplastis apyrenoidosa*, *Flintiella sangulnaria* of Rhodophyta and diverse Euglenozoa species) and nuclear-encoded maturases (13 spp. of Viridiplantae). The presence of alpha-proteobacteria in the basal clade of MT clade-3 suggests that mitochondrial group II IEPs originated from alpha-proteobacteria *via* mitochondrial endosymbiosis and were later transferred to the plastid or nuclear genome.

### Horizontal gene transfer from fungi and stramenopiles

A distinctive feature of the MT clade is the close relationship between sequences from fungi and stramenopiles (purple and orange colors, respectively, in Fig. 3a). For example, clade-2, -4, -6, -7, and -8 include fungal group II IEPs found in diverse genes, while clade-3, -5, -6, -7, and -8 include group II IEPs of stramenopiles, particularly from three diatom species (*Psammoneis japonica*, *Halamphora calidilacuna*, and *Halamphora coffeaeformis*, marked with white dots). Symbiotic relationships between algae and fungi have been reported. A well-known case includes 22 lichen-symbionts in the Trebouxiophyceae [40] and *Nanochloropsis oceanica*, which are internalized within hyphae of the fungus *Mortierella elongata* [41]. These symbiotic relationships begin with physical contact between fungal and algal cells, or the exchange of nutrients such as carbon and nitrogen. Both fungi and algae are physiologically active during co-cultivation, and eventually, the photosynthetic algae are internalized and function within fungi. Fungal group II introns can move vertically *via* the host mitogenome, by a fungal mitochondrial plasmid, or can be transferred horizontally across inter- and intraspecific boundaries [42, 43]. Although there are no existing reports of a symbiotic relationship between *Porphyridium* and fungi, our phylogenetic analysis (Fig. 3a) shows a close relationship between fungi-Bangiophyceae (clade-2, -7) and fungi-*P. aerugineum* (clade-4), suggesting intimate contact between these taxa.

In the case of stramenopiles, several diatom species, in particular *P. japonica* and *H. calidilacuna*, are closely related to sequences in *Porphyridium* species (clade-3, -5, -8) and to the Bangiophyceae and Florideophyceae (clade-6, -8). Mitogenome size in *H. calidilacuna* is 103 kb, which is the largest among diatom species known to date and includes a large number of group II introns [44]. A study of group II introns in other diatom species shows their transfer between non-diatom (*Chattonella*, Raphidophyceae) and diatom species [45, 46]. There are many reports of epiphytic diatoms inhabiting the surface of diverse algae (Rhodophyta, Chlorophyta, and Streptophyta) [47–49], where they exchange various substances such as inorganic nutrients [50]. The driving force of gene transfer between different species can be a plasmid or a virus. Plasmids carry foreign DNA and evidence of these genetic elements have been found in many algae and diatoms [51, 52]. Likewise, viruses can encode a large amount of DNA, and there are reports that HGT occurs in algae and diatoms *via* a virus [53, 54]. Although there are no reports of a symbiotic relationship between *Porphyridium*, diatoms, and fungi, our study suggests that HGTs may occur between these lineages.

**Fig. 4:**
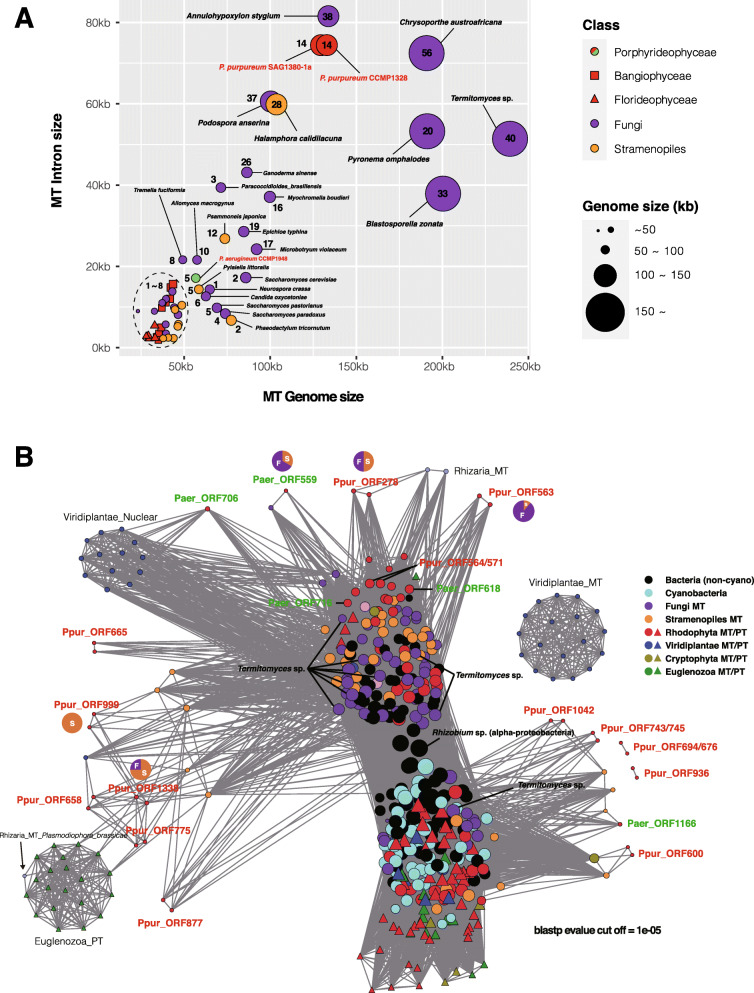
Identification of the major eukaryotic lineages related to *Porphyridium* mitogenome and group II intronic ORFs done using network analysis. **a** Dot plot comparing mitogenome size and intron size of species most closely related to *Porphyridium* mitogenome among the eukaryotes used in Fig. 3a. The number of IEPs is shown inside or next to the circle. **b** Protein similarity network of group II IEPs using all of the taxa shown in Fig. 3a, assembled using a BLASTp search (*e*-value cutoff = 1.0*e*^−5^). Nodes representing genes with high intramodular connectivity appear larger in the network. Node colors are different for each lineage (see legend)

To understand the interrelationships of group II intronic ORFs, we used the EGN [55] to reconstruct a network of all organisms used in Fig. 3a phylogenetic tree (*e*-value ≤ 1.0*e*^−5^, hit identity ≥ 20%). Two nodes were connected by an edge if they shared homologous DNA. The layout was produced by Cytoscape [56], using an edge-weighted spring-embedded model, whereby genomes sharing more DNA families are in closer proximity [57]. The gene network is divided into two subgroups, based on the group II intron of *Rhizobium* sp. (alpha-proteobacteria), the largest hub node: fungi-rich cluster (top cluster) and cyanobacteria-rich cluster (bottom cluster). In the fungi-rich cluster, most of the IEPs are located in *cox1* and *cob*, whereas in the cyanobacteria-rich cluster, most of the IEPs are located in rRNA and *psa*/*psb* genes. We find that IEPs transfer to specific genes in each organelle. Interestingly, there are many IEPs of *Halamphora calidilacuna* around these two subgroups and many IEPs of *Porphyridium* that are only connected to *H. calidilacuna*. Based on this, it can be expected that many IEPs of *Porphyridium* form a closer relationship with *H. calidilacuna* than with the two subgroups. Additionally, there are several clusters separated from the subgroups ('Viridiplanate_Nuclear', 'Viridiplantae_MT', 'Euglenozoa_PT'), and these clusters are expected to diverge from the biggest hub node due to the accumulation of genetic changes after group II intron transfer. Among them, the cluster of nuclear IEPs in Viridiplantae (i.e., 'Viridiplantae_Nuclear') appears to be a spliceosomal intron that exists in nuclear DNA. Therefore, the close relationship between *Porphyridium*, *H. calidilacuna*, and fungi is corroborated by the phylogenetic tree shown in Fig. 3a and the gene network (Fig. 4b and Additional file 1: Fig. S3b).

The network shows *Termitomyces* sp. to be a key species that includes 10 major hubs of group II intron IEPs (Fig. 4b). This fungal species has the largest mitogenome (239 kb, intron size: 51 kb), however, total intron size is fifth largest followed by *Annulohypoxylon styglum* (133 kb, intron size: 81 kb), *Chrysoporthe austroatricana* (190 kb, intron size: 72 kb), *Podospora anserina* (100 kb, intron size: 60 kb) and *Pyronema omphalodes* (191 kb, intron size: 53 kb) (Fig. 4a). In addition, we tested the correlation between total intron length, the number of IEPs, and mitogenome size among the taxa we investigated (Additional file 1: Fig. S1b). These results show that these three factors are moderately correlated with each other, suggesting that larger introns contain a larger number of IEPs. The gene network analysis was used to address the ambiguous topology (clade 5-2 in Fig. 3a) characterized by low bootstrap values (i.e., *orf278*, *orf1338*, *orf999*, *orf559*, and *orf563*) in the phylogenetic tree. As a result, four IEPs (i.e., *orf278*, *orf1338*, *orf559*, and *orf563*) except for *orf999*, which have node-edge relationships only with stramenopiles, form node-edge relationships with both fungi and stramenopiles. These results suggest multiple gene transfers from diatom and fungal species (Fig. 4b). This phenomenon has previously been reported [6] from cyanobacteria to *Euglena* [58], from unknown taxa to cryptophytes [59] or green algae [60], and between diatoms and *Chattonella* (Raphidophyceae) [45].

### Gene-specific vertical inheritance

Another interesting feature of the MT clade is that mitochondrial group II introns are predominantly encoded in the *cox1* gene (i.e., 103/162 in *cox1*, see Fig. 3c and Additional file 2: Table S4). Intron-rich *cox1* genes have been reported in diverse species [61, 62]; therefore, we hypothesized that there may be a target site in *cox1* that is targeted by group II introns, including a conserved splice donor and accepter site (i.e., 5′-GU----AG-3′) [63, 64]. To test this idea, we aligned a 10 bp region that is 5’ and 3’of the intron insertion site, as well as 5 bp of each terminus of the intron sequence for all taxa that were included in Fig. 3a and *Porphyridium* species only (Additional file 1: Fig. S4). In the intron region, there was a slightly elevated AG signal at the acceptor site (A 38%, G 48%) but no GU skewness (G 31%, T 34%) in all taxa, whereas in the *Porphyridium*-only alignment, a higher GU signal (G 42%, T 42%) was found at the donor site, but no AG signal at the acceptor site (A 37%, G 44%). There were no dominant sequences around splice sites in the *cox1* exon region, presumably due to the AT-rich mitogenome, even though the 5′ site 1 shows a dominant T (66%) (Additional file 1: Fig. S4). Rather than having a conserved insertion site, *cox1*, which is the most conserved gene in the mitogenome [65], likely provides a stable target for autocatalytic intron spread in populations and species. From a phylogenetic perspective, *orf559* and *orf563* in the *cox1* gene (clade 5-2, see Fig. 3a) form a well-supported monophyletic clade, suggesting vertical inheritance between *P. purpureum* and *P. aerugineum* (Fig. 3b). In addition to the *cox1* gene, group II IEPs are also present in other genes (Additional file 1: Fig. S3b). Most of the IEPs in the fungus-rich cluster are located in *cox1* and *cob*, whereas in the cyanobacterium-rich cluster, they are in mitochondrial rRNA and photosystem I and II genes in plastid genomes. These data suggest that IEPs were transferred from alpha-proteobacteria *via* mitochondrial primary endosymbiosis and targeted *cox1* and *cob* genes, whereas IEPs transferred from cyanobacteria and other proteobacteria were inserted in rRNA and photosystem I and II genes.

### Self-Proliferation

Four twintrons (*cobi858g2ii2121g2*, *cox1i387g2ii1392g2*, *atp9i87g2ii1464g2*, *cobi444g2ii1128g2*) and one twintron (*cox1i447g2ii1767g2*) were found in the *P. purpureum* and *P. aerugineum* mitogenomes, respectively (Additional file 1: Fig. S5)*.* Twintrons (introns-within-introns) are rarely reported, but interestingly, the first red algal twintrons were reported from the plastid genome of *P. purpureum* CCMP1328 [18]. Although these twintrons were found in the same strain within the plastid and mitochondrial DNA, they did not share sequence similarity. Instead, mitochondrial twintrons share sequence similarity (*e*-value ≤ 1*e*−10) to other introns in mitogenomes. For example, the twintron (*cobi858g2ii2121g2*) of *P. purpureum* shows high sequence similarity to other twintron (*atp9i87g2ii1464g2*), intron regions (*cobi777g2*, *cox1i1045g2*), and partial intronic ORFs (*orf563* in *cox1i387g2*, *orf999* in *nad5i750g2*), whereas a twintron in *orf1166* (*cox1i447g2ii1767g2*) shows sequence similarity to intron region (*cobi441g2*) and partial intronic ORF (*orf706* in *cox1i963g2*) (Additional file 1: Fig. S5). Organelle genome twintrons have been reported in the *cox1* gene of Lycopodiaceae species [37], as well as in the chloroplast genome of *Euglena* [66]. These results suggest that some group II introns were duplicated within a mitogenome, resulting in twintrons.

## Conclusions

**Fig. 5:**
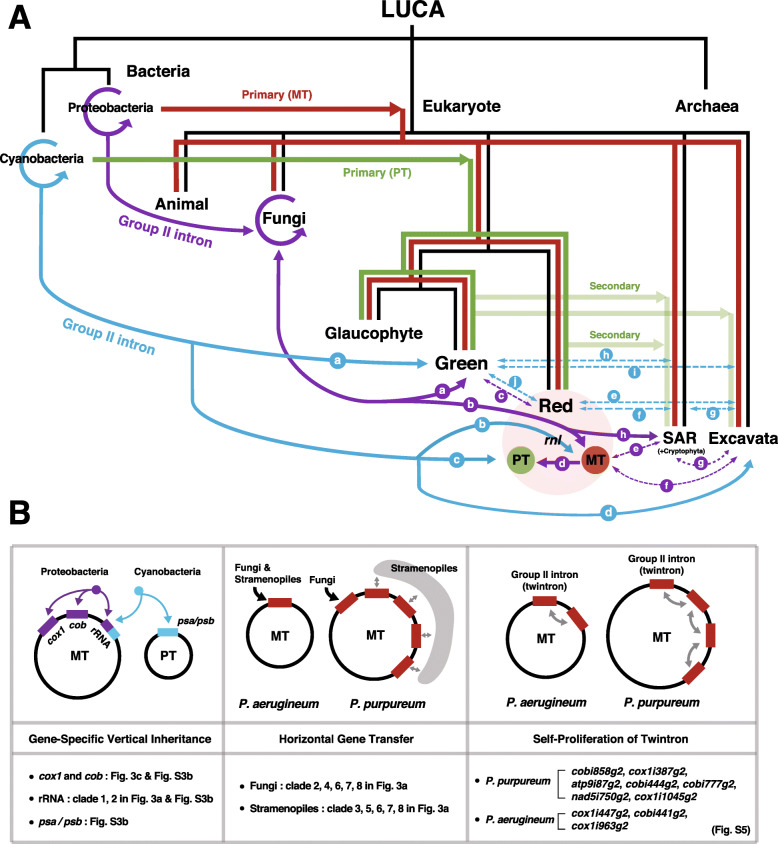
Hypothetical framework for the origin and evolution of group II introns and putative models to explain size expansion of *Porphyridium* mitogenomes. **a** Shown here is the evolutionary trajectory of group II introns in the tree of life. The proteobacterial primary endosymbiosis is shown with the red line and the cyanobacterial primary endosymbiosis with the green line. Secondary endosymbiosis involving green and red algae are shown by the lighter green lines. The movement of group II introns is shown in different colors (from cyanobacteria: blue; from proteobacteria: purple). Each colored circle containing a letter corresponds to the circles (colored and in alphabetical order) in the phylogenetic tree shown in Fig. 3a (cyanobacteria: blue-circles; proteobacteria: purple-circles). **b** This image shows three possible models (gene-specific vertical inheritance, horizontal gene transfer, and self-proliferation) to explain the spread of group II introns in *Porphyridium* mitogenomes

This study reports the largest red algal mitochondrial genomes described thus far. The unusual size expansion of *Porphyridium* mitogenomes is explained by the invasion of group II introns in genic regions and the expansion of repeats in the large intergenic region. Our work makes clear that group II introns are able to transfer across species boundaries. A model that depicts the complex evolutionary trajectory of group II introns is shown in Fig. 5. We find that although the two strains of *P. purpureum* that were studied (i.e., CCMP1328 and SAG1380-1a) contain the same set of introns and IEPs, their genome sizes differ widely (132 kbp vs. 129 kbp) due to repeat content (23 kbp vs. 18 kbp, respectively), demonstrating the dynamic nature of red algal mitogenome evolution. Although *P. purpureum* and *P. aerugineum* are sister species, the genome size, number of introns (i.e., IEPs), and length of LIRs are substantially different between these taxa (Fig. 1a). In this case, intron expansion in *P. purpureum* (74 kbp) was a major contributor to the genome size difference when compared to *P. aerugineum* (17 kbp of introns), which likely occurred post-speciation. When comparing the two species of *Porphyridium*, only *orf559* and *orf563* in *cox1* were likely to have been present in their common ancestor, with the remainder being derived from more recent HGT events or twintron formation (Fig. 1b). As such, the three *Porphyridium* species provide a model for understanding the forces driving organelle genome expansion and the mobility of group II introns.

Based on our data, we demonstrate three possible scenarios for group II intron spread: gene-specific vertical inheritance, HGT, and self-proliferation (Fig. 5). Because most eukaryotes share group II introns located in a few conserved genes (i.e., *cox1*, *cob*, rRNA, and PSI/PSII subunit genes), it is likely that many have been inherited vertically since the primary endosymbiosis events involving proteobacteria (mitochondrion) and cyanobacteria (plastids). Group II introns in *cox1*, *cob*, and some rRNA genes may be derived from alpha-proteobacteria, whereas group II introns in PSI/PSII subunit genes and some rRNA genes were derived from cyanobacteria and other bacteria (Fig. 3). The cyanobacterium-derived rRNA introns later invaded the mitochondrial genome (clade-1 in Fig. 3a). Several fungal and diatom species are key players in these mobility events across species/phylum boundaries. In addition, there are gene transfers between organelles (e.g., *Bulboplastis apyrenoidosa*, *Flintiella sangulnaria* of Rhodophyta and diverse Euglenozoa) as well as from organelles to the nucleus (some Viridiplantae species, see Additional file 1: Fig. S3b). The large-scale protein similarity-based network analysis of IEPs resolves two connected components, each of which contains plastid and mitochondrion encoded mobility enzymes present in distantly related species. These components are linked by bacterial IEPs. The network results suggest that organelle DNA and bacterial genomes provide “safe harbors” for autocatalytic RNAs that facilitate their spread. These mobile elements are likely continually being purged from extant nuclear genomes due to negative impacts on RNA stability and translation [13].

Although it remains highly challenging to account for the entirety of group II intron origin and evolution, novel genome data such as from *Porphyridium*, provide a more accurate picture of the dynamic history of these mobile genetic elements. Our work underlines the need to more deeply sample intron-rich clades to reconstruct intron origins and losses rather than comparing these sequences across vast phylogenetic distances with no understanding of the impact of recent evolutionary events. Another consideration that our study points to is the possibility that group II introns are “canaries in the coal mine” for HGTs of non-mobile protein coding genes. If proximity fosters HGT, as is generally accepted, then the patterns of transfer shown here may be useful tools for searching for other HGT events between lineages that share homologous group II introns.

## Methods

### Strain information and sample preparation

Three strains of *Porphyridium* [*Porphyridium purpureum* CCMP1328, *P. purpureum* SAG1380-1a, *P. aerugineum* CCMP1948] were obtained from culture collections (NCMA, https://ncma.bigelow.org/; SAG, https://www.uni-goettingen.de/). Each strain was sub-cultured in L1-Si standard medium, modified brackish DY-V medium, and DY-V standard medium [67], respectively.

### Genome sequencing, assembly, gene prediction, and annotation

Cells were harvested by centrifugation (14,000 rpm for 5 min). Total genomic DNA was extracted using the CTAB method [68]. Total DNA from each species was sequenced using ONT (Oxford Nanopore Technologies, Oxford, England) and Illumina (NovaSeq 6000 system: paired-end TruSeq Nano DNA Prep Kit) platforms. Each sample for ONT sequencing was prepared as following the Nanopore library preparation protocols provided by the manufacturer. The ONT library was constructed by using Ligation Sequencing Kit (SQK-LSK109). This library was sequenced with Ligation Sequencing Kit (SQK-LSK109), Flow Cell Priming Kit (EXP-FLP001), and Flow Cell (FLO-MIN106). All runs were performed on the GridION sequencer. We assembled mitochondria genome using the Canu assembler v1.4 [69]. Mitogenome-related contigs were sorted by customized Python scripts with local BLAST programs compared to the reference genome data (*Bangia fuscopurpurea*; GenBank accession NC_026905.1), and the sorted contigs were re-assembled to construct consensus mitochondrial genomes. The Illumina data were then mapped against the Canu assembly contigs using CLC Genomics Workbench v8.0.3 (CLC Bio., Aarhus, Denmark) for error correction.

Gene prediction was carried out with a comparison to the reference gene sequences using BLASTx search (*e*-value ≤ 1*e*−10) and the Geneious 8.1.2 program (Kearse et al. 2012) with translation_table 4 (Mold Protozoan Mitochondrial). To predict tRNA regions, we used ARAGORN [70] using the default option. To predict rRNA regions, RNAmmer 1.2 Server [71] was used. BLASTn search was used to check the rRNA region manually. The twintrons present in the *Porphyridium* mitogenomes were aligned and compared to other introns using BLASTn to allow identification of outer and inner introns. We used the previously proposed nomenclature [37] to discriminate between the *Porphyridium* mitochondrial introns. Finally, the mitochondria genome map was visualized with the GenomeVx program [72].

### Analysis of repeat sequences in mitochondrial genomes

Repeats were identified by BLASTn, dot-plot comparison in a genomic similarity search tool (YASS) [22], and ETANDEM tool (EMBOSS package) [73]. The input size for searching tandem repeat using ETANDEM tool was divided into 2~100 bp, 101~200 bp, 201~300 bp, 301~400 bp, and 401~500 bp. All the settings of YASS and ETANDEM were default values.

### Gene network and phylogenetic analysis of mitochondrial intronic ORF

To identify the relationship between organelle intronic ORFs and nuclear genome of *Porphyridium purpureum* CCMP1328 (NCBI BioProject ID: PRJNA560054, Genome accession number VRMN00000000), all intronic ORFs in the mitochondria and chloroplast genomes of three *Porphyridium* species were used. To construct phylogenetic trees, homology searches for mitochondrial intronic ORF were conducted against local nr database using BLASTp (*e*-value cutoff=1e^−05^, word size=6). The collected homologous genes were aligned using MAFFT v7.313 (default option: --auto) and phylogenetic relationships were inferred with IQ-tree (v1.6.7) (model test: -m TEST and replications: -bb 1000) [74]. To avoid biased taxon sampling, the search criterion used was the maximum number of taxa for each query (i.e., max phylum limitation = top 75 species), thereafter, duplicated taxa were removed. In addition, we removed short sequences (under 175 amino acids) from the phylogenetic analysis, with further filtering by selecting a single representative from each monophyletic clade. The gene network analysis was done using Cytoscape [56] and the EGN program [55].

## Supplementary Information

**Additional file 1: Figures S1-S5.** **Fig. S1.** Correlation analysis. (a) Correlation analysis between mitochondria genome size and length of CDS+ORF, intergenic region, and intron. The black dots represent all published red algal mitogenomes in NCBI, whereas colored dots represent three *Porphyridium* species. (*P. purpureum* CCMP1328; Red, *P. purpureum* SAG1380-1a; Yellow, *P. aerugineum* CCMP1948; Green). Blue lines indicate linear regression line, black dot lines indicate prediction interval, and grey bounds indicates confidence interval with 95% confidence level. (b) Correlation analysis between mitochondria genome size, length of intron, and number of IEP. Blue lines indicate linear regression line, red dot lines indicate prediction interval, and grey bounds indicates confidence interval with 95% confidence level. **Fig. S2.** Repeat distribution of *Porphyridium* and red algal mitogenome. (a) Frequency of repeats by length in the *Porphyridium* mitogenome. The cutoff value for tandem repeats was 100 bp. (b) Genome size versus the number of repeats in mitogenome among the red algae. (c) Repeat density in the red algae (number of repeat / 1 kbp). (d) Frequency of repeats by length in the red algal mitogenome. The cutoff value for tandem repeats was 100 bp. (e) Information on the taxa used in Figures S2b-d. **Fig. S3.** Gene network analysis. (a) Similarity network generated from all three *Porphyridium* mitochondrial ORFs against BLAST similarity search to the plastid and nuclear genome of *P. purpureum* CCMP1328. Each colored node (red, orange, yellow, blue, green, and purple) represents an ORF in three genomes including mitochondria, plastid, and nuclear. Each edge (line) represents a significant HSP (high scoring segment pair) according to *p* value 1e-0.5. (b) Group II IEPs network analysis using whole data in Figure 3a phylogenetic tree. Gene network of group II IEPs based on BLASTp (*e*-value ≤ 1.0*e* ^-5^, hit identity ≥ 20%). All group II IEPs in Figure 3a phylogenetic tree were used as queries, and the dataset required for gene network was constructed using EGN software. The gene network was produced by Cytoscape using an edge-weighted spring-embedded model. Nodes representing genes with high intramodular connectivity appear larger in the network. The more DNA families a node shares, the closer the distance is. Node colors are displayed differently for each gene. **Fig. S4.** Intron insertion site analysis in *cox1* gene. Partial sequences of exon and intron near the splice site were aligned to find common features for the intron insertion site of the *cox1* gene. In the exon region, 10 bp of each 5’ and 3’-end were aligned by MAFFT alignment program. In the case of intron region, 5 bp of each 5’ and 3’-end were aligned. A sequence logo was added to the panel below each alignment to indicate dominant sequences. (a) Results from all taxa and (b) results from *Porphyridium*-only analysis. **Fig. S5.** Location of twintrons in *Porphyridium* mitogenome. Location of twintrons is presented based on the results of BLASTn search and ClustalW alignment. The *e*-value cutoff is 1*e*-10. In each gene, exons are shown in yellow, introns in white, intronic ORFs in green, and twintrons in gray with dotted lines. Based on the BLASTn results, the areas sharing similarity are shown by connecting them with the red color. The nomenclature of introns and twintrons follows the previously proposed system [37].

**Additional file 2: Tables S1-S5. Table S1.** Red algal mitogenome information. The genome size, GC content, CDS, ORF, and intron information of red algal mitogenomes published in NCBI are shown in this table. **Table S2.** Molecular organization of the mitochondrial genes in red algae. The composition of mitochondrial genes of red algal mitogenomes published in NCBI is shown in this table. **Table S3.** Mitogenome information of *Porphyridium* species. The length of gene and CDS, number of group II IEPs, and the proportion of introns in each mitochondrial gene of three *Porphyridium* species are shown in this table. **Table S4.** Distribution of mitochondrial group II introns in Figure 3. This table shows the number of IEPs in mitochondrial genes in all eukaryotic taxa used in the phylogenetic tree in Figure 3. **Table S5.** Public dataset used in this study. This table shows the GenBank accession numbers and references for the mitogenomes used in this study.

**Additional file 3.** Alignment file (NEXUS format): Alignment file for Fig. 3a (NEXUS format)

## Data Availability

The data was deposited in the GenBank under the accession numbers of MT483995 [75] (*P. aerugineum* CCMP1948), MT483996 [76] (*P. purpureum* CCMP1328), and MT483997 [77] (*P. purpureum* SAG1380-1a). All data information used in this study are included in Additional file 2: Table S5. Alignment file used in this study is included in Additional file 3.

## Abbreviations

Mitogenome: Mitochondrial genome
LIR: Large intergenic region
IEP: Intron-encoded protein
ORF: Open reading frame
snRNA: Small nuclear RNA
LTR: Long terminal repeat
CDS: Coding sequence
EGT: Endosymbiotic gene transfer
HGT: Horizontal gene transfer
MT: Mitochondria
PT: Plastid
PSI: Photosystem I
PSII: Photosystem II
CTAB: Cetyltrimethylammonium bromide
ONT: Oxford Nanopore Technologies
