# Supplementary material for: Group II intron and repeat-rich red algal mitochondrial genomes demonstrate the dynamic recent history of autocatalytic RNAs
Source: BMC Biol. 2022 Jan 7;20:2. doi: 10.1186/s12915-021-01200-3 (PMC8742464; doi:10.1186/s12915-021-01200-3)
Supplement: Supplementary file 1 — Additional file 1: Figures S1-S5. Fig. S1. Correlation analysis. (a) Correlation analysis between mitochondria genome size and length of CDS+ORF, intergenic region, and intron. The black dots represent all published red algal mitogenomes in NCBI, whereas colored dots represent three Porphyridium species. (P. purpureum CCMP1328; Red, P. purpureum SAG1380-1a; Yellow, P. aerugineum CCMP1948; Green). Blue lines indicate linear regression line, black dot lines indicate prediction interval, and grey bounds indicates confidence interval with 95% confidence level. (b) Correlation analysis between mitochondria genome size, length of intron, and number of IEP. Blue lines indicate linear regression line, red dot lines indicate prediction interval, and grey bounds indicates confidence interval with 95% confidence level. Fig. S2. Repeat distribution of Porphyridium and red algal mitogenome. (a) Frequency of repeats by length in the Porphyridium mitogenome. The cutoff value for tandem repeats was 100 bp. (b) Genome size versus the number of repeats in mitogenome among the red algae. (c) Repeat density in the red algae (number of repeat / 1 kbp). (d) Frequency of repeats by length in the red algal mitogenome. The cutoff value for tandem repeats was 100 bp. (e) Information on the taxa used in Figures S2b-d. Fig. S3. Gene network analysis. (a) Similarity network generated from all three Porphyridium mitochondrial ORFs against BLAST similarity search to the plastid and nuclear genome of P. purpureum CCMP1328. Each colored node (red, orange, yellow, blue, green, and purple) represents an ORF in three genomes including mitochondria, plastid, and nuclear. Each edge (line) represents a significant HSP (high scoring segment pair) according to p value 1e-0.5. (b) Group II IEPs network analysis using whole data in Figure 3a phylogenetic tree. Gene network of group II IEPs based on BLASTp (e-value ≤ 1.0e -5, hit identity ≥ 20%). All group II IEPs in Figure 3a phylogenetic tree were used as q [file 12915_2021_1200_MOESM1_ESM.pdf]

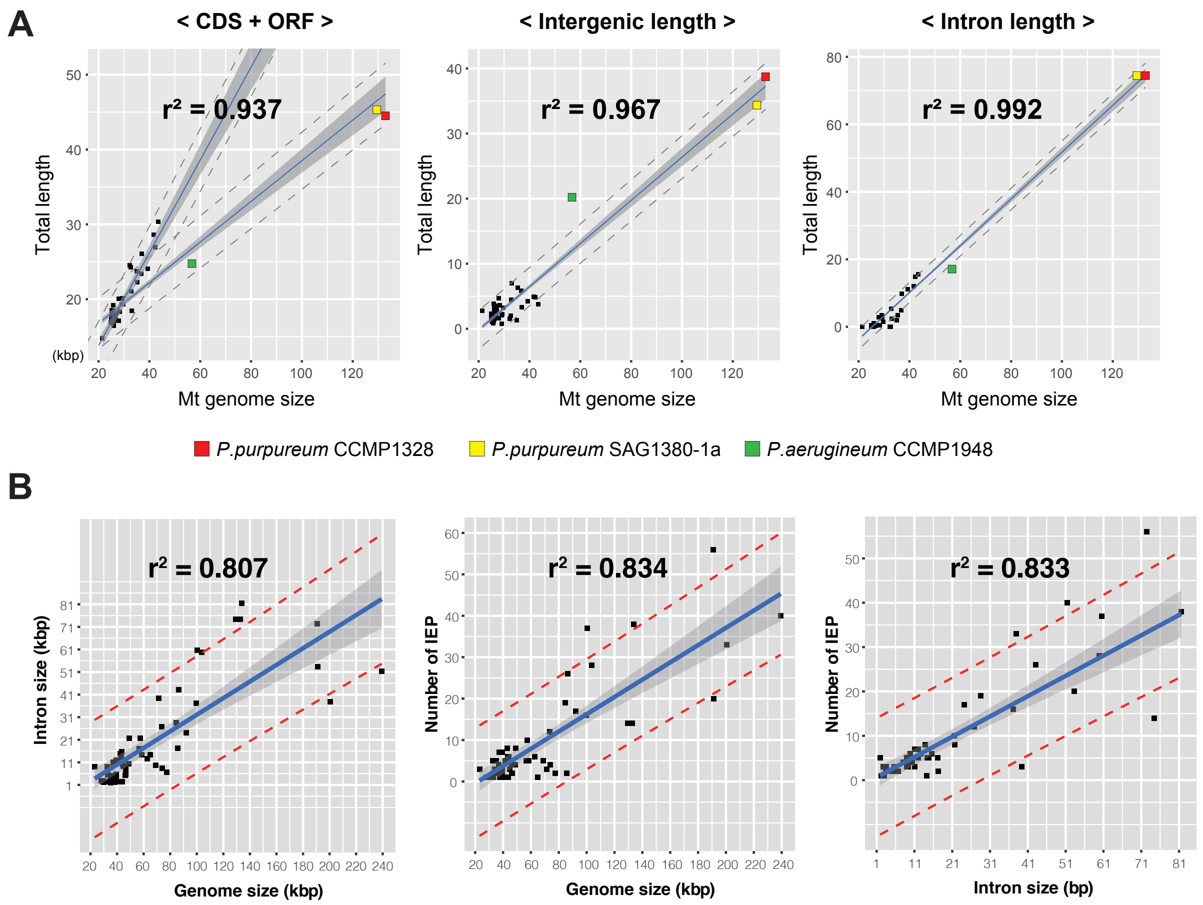

Fig. S1 Correlation analysis. (**a**) Correlation analysis between mitochondria genome size and lengths of CDS+ORF, intergenic regions, and introns. The black dots represent all published red algal mitogenomes in NCBI, whereas colored dots represent three *Porphyridium* species. (*P. purpureum* CCMP1328; Red, *P. purpureum* SAG1380-1a; Yellow, *P. aerugineum* CCMP1948; Green). Blue lines indicate linear regression line, black dot lines indicate prediction interval, and grey bounds indicates confidence interval with 95% confidence level. (**b**) Correlation analysis between mitochondria genome size, length of introns, and number of IEPs. Blue lines indicate linear regression line, red dot lines indicate prediction interval, and grey bounds indicates confidence interval with 95% confidence level.

A

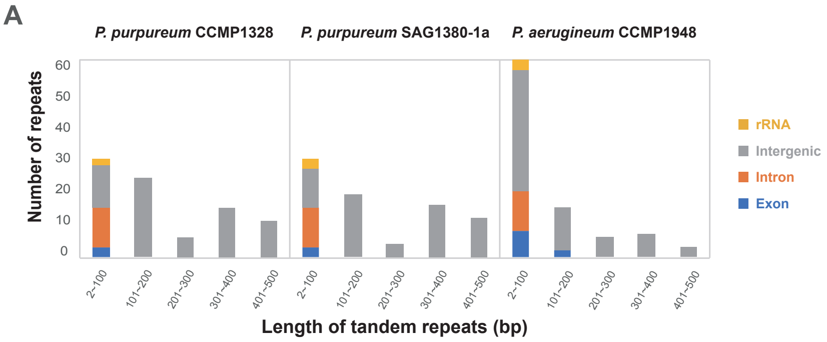

B

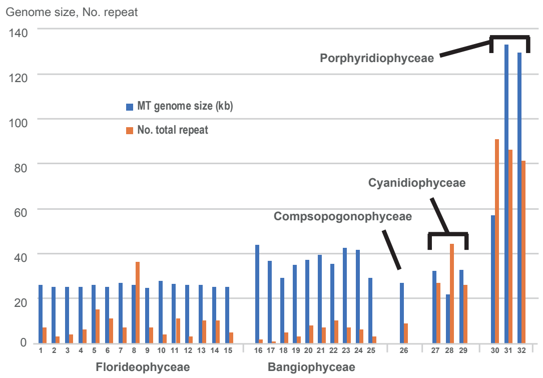

D

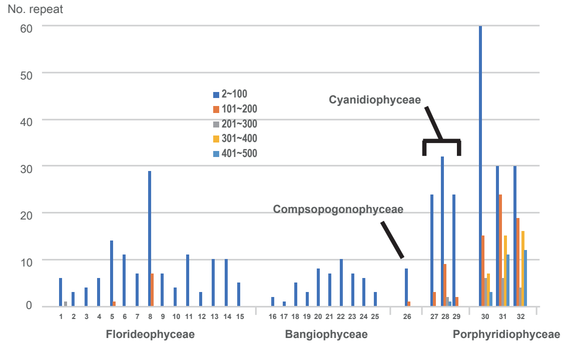

C

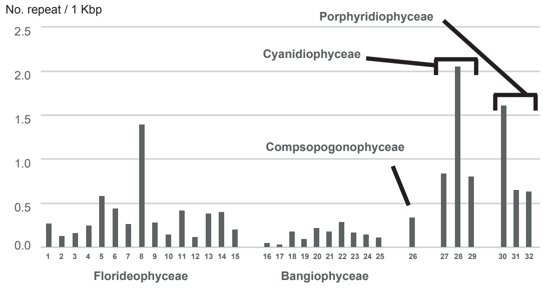

E

**Florideophyceae (15/67)**	*(1)Gracilaria vermiculophylla* *(2)Kappaphycus alvarezii* *(3)Gelidium galapagense* *(4)Gelidium sinicola* *(5)Chondrus crispus* *(6)Gracilariophila oryzoides* *(7)Gracilariopsis andersonii* *(8)Plocamiocolax pulvinata* *(9)Gelidium vagum* *(10)Grateloupia angusta* *(11)Gracilariopsis chorda* *(12)Rhodymenia pseudopalmata* *(13)Sporolithon durum* *(14)Gracilaria salicornia* *(15)Kappaphycus striatus*
**Bangiophyceae (10)**	*(16)Bangia fuscopurpurea* *(17)Porphyra purpurea* *(18)Porphyra umbilicalis* *(19)Pyropia fucicola* *(20)Pyropia haitanensis* *(21)Pyropia kanakaensis* *(22)Pyropia nitida* *(23)Pyropia tenera* *(24)Pyropia yezoensis* *(25)Wildemania schizophylla*
**Compsopogonophyceae (1)**	*(26)Compsopogon caeruleus*
**Cyanidiophyceae (3)**	*(27)Cyanidioschyzon merolae* *(28)Galdieria sulphuraria* *(29)Cyanidiaceae sp. MX-AZ01*
**Porphyridiophyceae (3)**	*(30)Porphyridium aerugineum CCMP1948* *(31)Porphyridium purpureum CCMP1328* *(32)Porphyridium purpureum SAG1380-1a*

Fig. S2 Repeat distribution of *Porphyridium* and red algal mitogenomes. (**a**) Frequency of repeats by length in the *Porphyridium* mitogenome. The cutoff value for tandem repeats was 100 bp. (**b**) Genome size versus the number of repeats in mitogenomes among the red algae. (**c**) Repeat density in the red algae (number of repeat / 1 kbp). (**d**) Frequency of repeats by length in the red algal mitogenomes. The cutoff value for tandem repeats was 100 bp. (**e**) Information on the taxa used in Figures S2b-d.

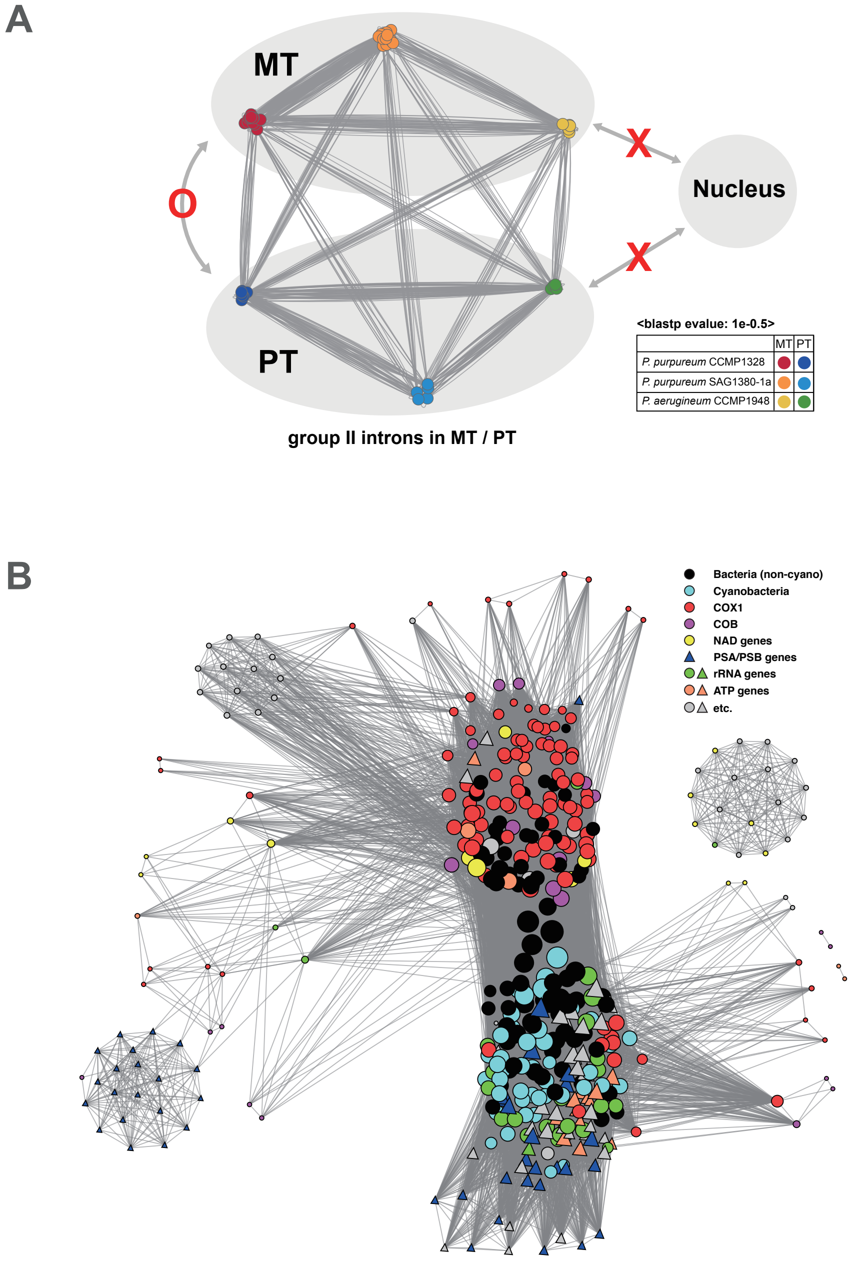

Fig. S3 Gene network analysis. **(a)** Similarity network generated from all three *Porphyridium* mitochondrial ORFs against BLAST similarity search to the plastid and nuclear genome of *P. purpureum* CCMP1328. Each colored node (red, orange, yellow, blue, green, and purple) represents an ORF in three genomes including mitochondria, plastid, and nuclear. Each edge (line) represents a significant HSP (high scoring segment pair) according to *e*-value $1e^{-0.5}$. **(b)** Group II IEPs network analysis using whole data in Figure 3a phylogenetic tree. Gene network of group II IEPs based on BLASTp (*e*-value $\leq 1.0e^{-5}$, hit identity $\geq$ 20%). All group II IEPs in Figure 3a phylogenetic tree were used as queries, and the dataset required for gene network was constructed using EGN software. The gene network was produced by Cytoscape using an edge-weighted spring-embedded model. Nodes representing genes with high intramodular connectivity appear larger in the network. The more DNA families a node shares, the closer the distance is. Node colors are displayed differently for each gene.

< Intron insertion site analysis in *cox1* gene >

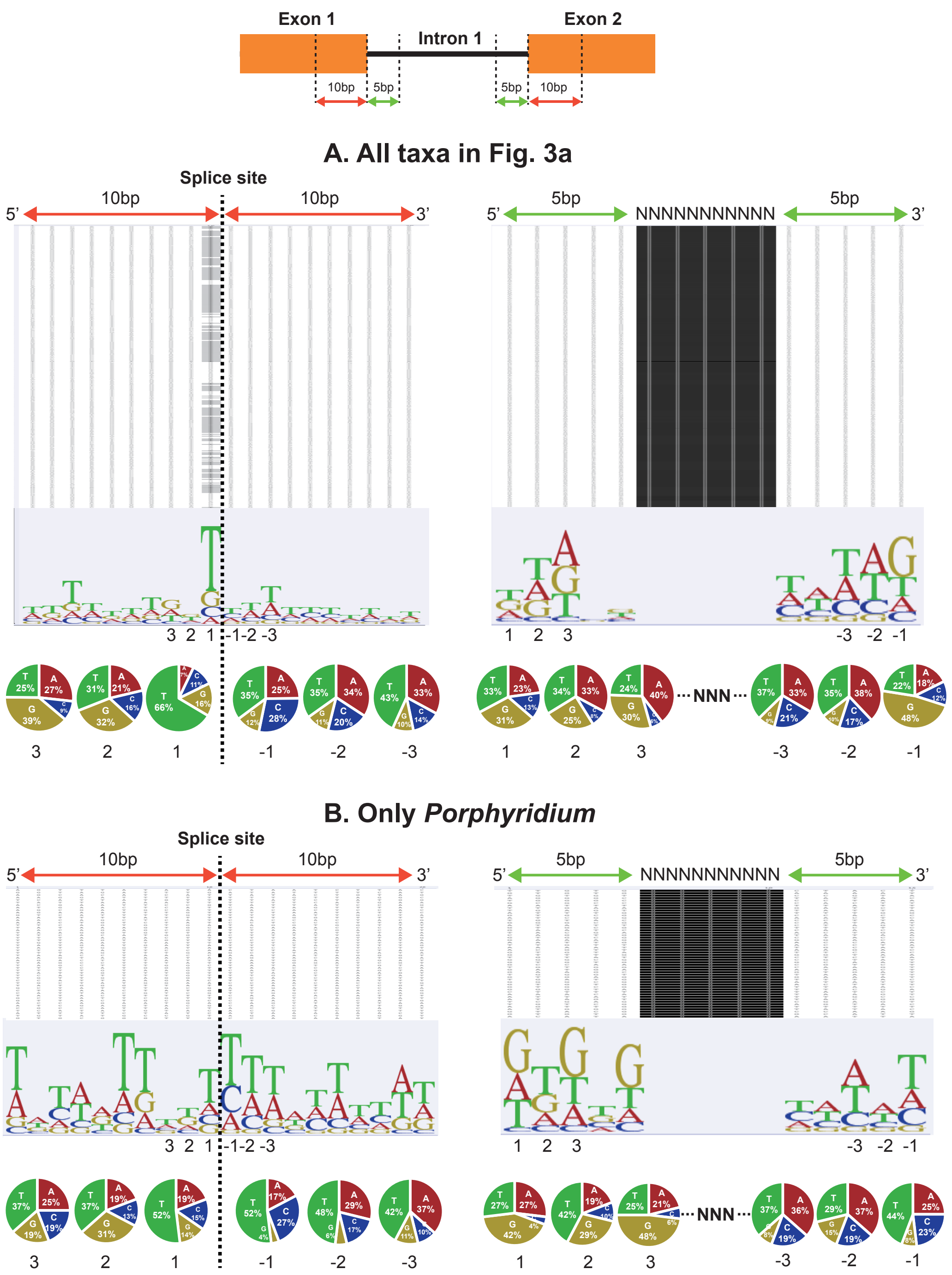

Fig. S4 Intron insertion site analysis in *cox1* gene. Partial sequences of exon and intron near the splice site were aligned to find common features for the intron insertion site of the *cox1* gene. In the exon region, 10 bp of each 5' and 3'-end were aligned by MAFFT alignment program. In the case of intron region, 5 bp of each 5' and 3'-end were aligned. A sequence logo was added to the panel below each alignment to indicate dominant sequences. **(a)** Results from all taxa and **(b)** results from *Porphyridium*-only analysis.

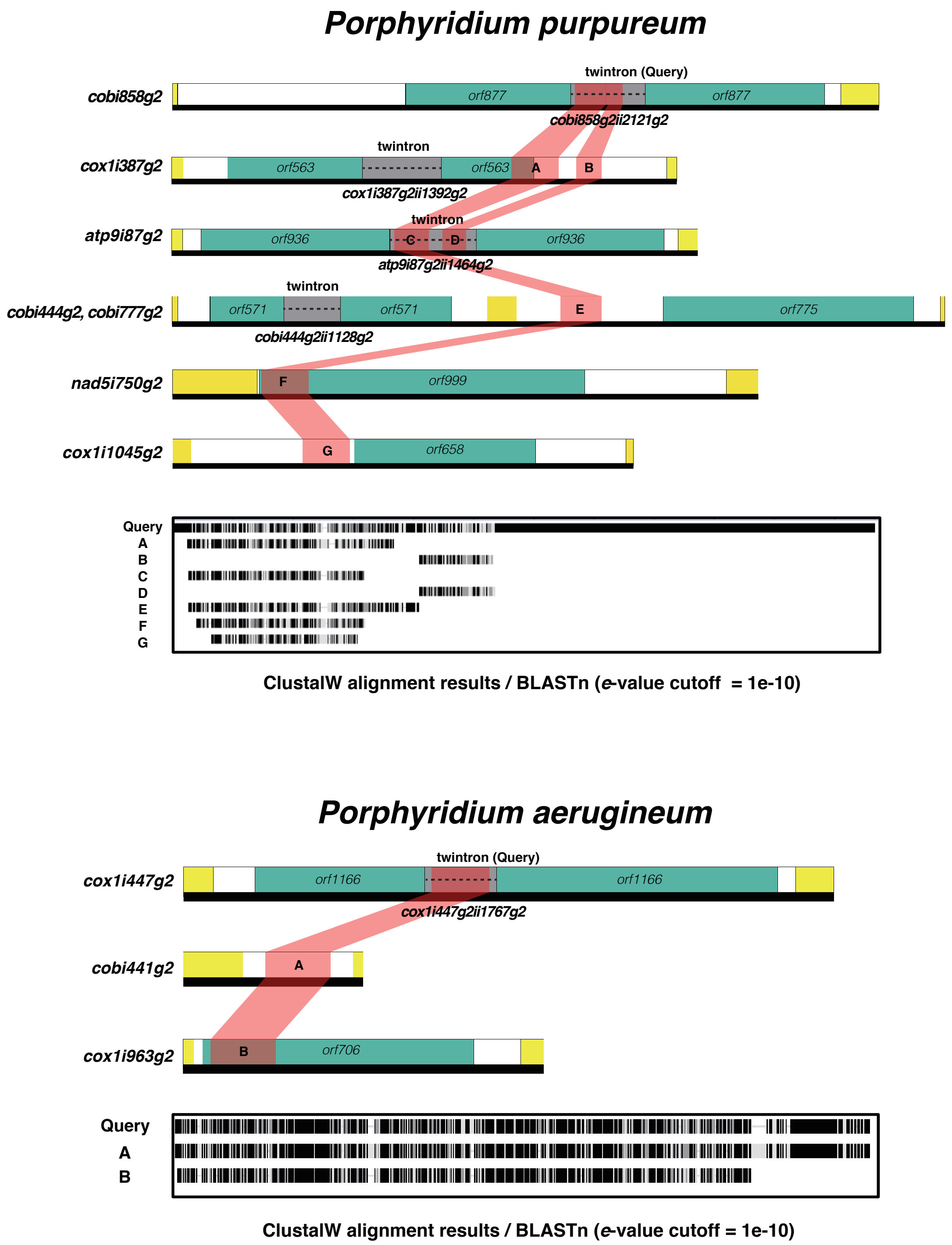

Fig. S5 Location of twintrons in *Porphyridium* mitogenome. Location of twintrons is presented based on the results of BLASTn search and ClustalW alignment. The *e*-value cutoff is $1e^{-10}$. In each gene, exon is shown in yellow, intron in white, intronic ORF in green, and twintron in gray with dotted line. Based on the BLASTn results, the areas having similarity are shown by connecting them in red color. The nomenclature of intron and twintron is followed by previously proposed nomenclature [64].
